# Contribution to the History of Medical Education and Medical Institution in the United States of America, 1776-1876

**Published:** 1878-02

**Authors:** 


*Contributions to the History of Medical Education and Medical Institution in the United States of America,* 1776–1876.

This is a Special Report prepared for the U. S. Bureau of Education by N. S. Davis, A. M., M. D., the Report on Public Libraries issued some months ago, being the first of the historical series intended to bring the progress of education in this Country down to the Centennial year. This work on medical education is the second subject. As there are many indications of the increase of public and professional interest in advancing the standard of medical education, this monograph will prove a valuable contribution to this purpose.

